# An intrinsically disordered proteins community for ELIXIR

**DOI:** 10.12688/f1000research.20136.1

**Published:** 2019-10-15

**Authors:** Norman E. Davey, M. Madan Babu, Martin Blackledge, Alan Bridge, Salvador Capella-Gutierrez, Zsuzsanna Dosztanyi, Rachel Drysdale, Richard J. Edwards, Arne Elofsson, Isabella C. Felli, Toby J. Gibson, Aleksandras Gutmanas, John M. Hancock, Jen Harrow, Desmond Higgins, Cy M. Jeffries, Philippe Le Mercier, Balint Mészáros, Marco Necci, Cedric Notredame, Sandra Orchard, Christos A. Ouzounis, Rita Pancsa, Elena Papaleo, Roberta Pierattelli, Damiano Piovesan, Vasilis J. Promponas, Patrick Ruch, Gabriella Rustici, Pedro Romero, Sirarat Sarntivijai, Gary Saunders, Benjamin Schuler, Malvika Sharan, Denis C. Shields, Joel L. Sussman, Jonathan A. Tedds, Peter Tompa, Michael Turewicz, Jiri Vondrasek, Wim F. Vranken, Bonnie Ann Wallace, Kanin Wichapong, Silvio C. E. Tosatto

**Affiliations:** 1 Division of Cancer Biology, Institute of Cancer Research, UK, London, SW3 6JB, UK; 2 MRC Laboratory of Molecular Biology,, Cambridge, CB2 0QH, UK; 3 Institut de Biologie Structurale, Université Grenoble Alpes, Grenoble, 38000, France; 4 Swiss-Prot Group, SIB Swiss Institute of Bioinformatics, Geneva, Switzerland; 5 Life Sciences Department, Barcelona Supercomputing Center (BSC), Barcelona, Spain; 6 Department of Biochemistry, Eötvös Loránd University, Budapest, H-1117, Hungary; 7 ELIXIR Hub, Wellcome Genome Campus, Cambridge, CB10 1SD, UK; 8 School of Biotechnology and Biomolecular Sciences, University of New South Wales, Sydney, NSW, Australia; 9 Department of Biochemistry and Biophysics and Science for Life Laboratory, Stockholm University, Stockholm, Sweden; 10 Department of Chemistry and CERM “Ugo Schiff”, University of Florence, Florence, Italy; 11 Structural and Computational Biology Unit, European Molecular Biology Laboratory, Heidelberg, Germany; 12 Protein Data Bank in Europe, European Bioinformatics Institute (EMBL-EBI), European Molecular Biology Laboratory, Cambridge, CB10 1SD, UK; 13 Conway Institute of Biomolecular & Biomedical Research, University College Dublin, Belfield, Dublin, D4, Ireland; 14 European Molecular Biology Laboratory, Hamburg, Germany; 15 Department of Biomedical Sciences, University of Padua, Padua, Italy; 16 Centre for Genomic Regulation (CRG), The Barcelona Institute of Science and Technology, Barcelona, 08003, Spain; 17 Universitat Pompeu Fabra (UPF), Barcelona, Spain; 18 European Bioinformatics Institute (EMBL-EBI), European Molecular Biology Laboratory, Cambridge, CB10 1SD, UK; 19 BCPL-CPERI, Centre for Research & Technology Hellas (CERTH), Thessalonica, 57001, Greece; 20 Institute of Enzymology, Research Centre for Natural Sciences of the Hungarian Academy of Sciences, Budapest, H-1117, Hungary; 21 Computational Biology Laboratory, Danish Cancer Society Research Center, Copenhagen, 2100, Denmark; 22 Bioinformatics Research Laboratory, Department of Biological Sciences, University of Cyprus, Nicosia, CY-1678, Cyprus; 23 HES-SO/HEG and SIB Text Mining, Swiss Institute of Bioinformatics, Geneva, Switzerland; 24 Department of Genetics, University of Cambridge, Cambridge, CB2 3EH, UK; 25 University of Wisconsin-Madison, Madison, WI, 53706-1544, USA; 26 Department of Biochemistry, University of Zurich, Zurich, Switzerland; 27 Department of Structural Biology and the Israel Structural Proteomics, Center (ISPC), Weizmann Institute of Science, Reḥovot, 7610001, Israel; 28 VIB Center for Structural Biology (CSB), VIB Flemish Institute for Biotechnology, Brussels, 1050, Belgium; 29 Faculty of Medicine, Medizinisches Proteom-Center, Ruhr University Bochum, GesundheitsCampus 4, Bochum, 44801, Germany; 30 Institute of Organic Chemistry and Biochemistry, CAS, Prague, Czech Republic; 31 VUB/ULB Interuniversity Institute of Bioinformatics in Brussels and Structural Biology Brussels, Vrije Universiteit Brussel, Brussels, B-1050, Belgium; 32 Institute of Structural and Molecular Biology, Birkbeck College, University of London, London, WC1H 0HA, UK; 33 Department of Biochemistry, Cardiovascular Research Institute Maastricht (CARIM), Maastricht University, Maastricht, The Netherlands

**Keywords:** ELIXIR, intrinsically disordered proteins, protein-protein interactions, protein function, databases, community standards, protein dynamics, cellular regulation

## Abstract

Intrinsically disordered proteins (IDPs) and intrinsically disordered regions (IDRs) are now recognised as major determinants in cellular regulation. This white paper presents a roadmap for future e-infrastructure developments in the field of IDP research within the ELIXIR framework. The goal of these developments is to drive the creation of high-quality tools and resources to support the identification, analysis and functional characterisation of IDPs. The roadmap is the result of a workshop titled “An intrinsically disordered protein user community proposal for ELIXIR” held at the University of Padua. The workshop, and further consultation with the members of the wider IDP community, identified the key priority areas for the roadmap including the development of standards for data annotation, storage and dissemination; integration of IDP data into the ELIXIR Core Data Resources; and the creation of benchmarking criteria for IDP-related software. Here, we discuss these areas of priority, how they can be implemented in cooperation with the ELIXIR platforms, and their connections to existing ELIXIR Communities and international consortia. The article provides a preliminary blueprint for an IDP Community in ELIXIR and is an appeal to identify and involve new stakeholders.

## Introduction

Intrinsically disordered regions (IDRs), protein segments that lack persistent secondary or tertiary structure ( Chouard, 2011; Dyson & Wright, 2005; Tompa, 2011), are predicted to cover almost a third of the residues in eukaryotic proteomes ( Pancsa & Tompa, 2012; Xue *et al.*, 2012). IDRs play a central role in cell regulation and contribute significantly to the cellular complexity of higher eukaryotes ( Dunker *et al.*, 2008; Dyson & Wright, 2005; Forman-Kay & Mittag, 2013; Gouw *et al.*, 2018; Mitrea & Kriwacki, 2016; Schad *et al.*, 2018; Tompa, 2005; Van Roey *et al.*, 2014). They represent a major source of protein diversity and versatility on the level of organisms and during evolution ( Babu *et al.*, 2012; Buljan *et al.*, 2012; Davey *et al.*, 2015; Light *et al.*, 2013; Weatheritt & Gibson, 2012). In the human proteome, IDRs are expected to contain up to one hundred thousand interaction interfaces and a million sites of post-translational modification ( Tompa *et al.*, 2014). However, to date, only a small fraction of these functional modules have been characterised ( Gouw *et al.*, 2018; Schad *et al.*, 2018).

IDR-mediated interactions are commonly found in dynamic and transient complexes that underlie enzyme inhibition, signal transduction and liquid-liquid phase transition ( Borgia *et al.*, 2018; Ivarsson & Jemth, 2019; Kriwacki *et al.*, 1996; Martin & Mittag, 2018; Mitrea & Kriwacki, 2016; Olsen *et al.*, 2017; Scott & Pawson, 2009; Wright & Dyson, 2015). IDR-mediated interactions are also major determinants of protein regulation, and are so far known to affect: (i) the post-translational modification state of a protein by acting as docking sites to recruit modifying enzymes; (ii) the cellular half-life of a protein by recruiting E3 ubiquitin ligases resulting in ubiquitin-dependent proteasomal degradation of the protein; and (iii) the localisation of a protein by acting as signals that target proteins to specific subcellular locations ( Beltrao *et al.*, 2012; Davey & Morgan, 2016; Gouw *et al.*, 2018; Guharoy *et al.*, 2016; Iakoucheva *et al.*, 2004; Mészáros *et al.*, 2017; Van Roey *et al.*, 2014). Many functions of IDRs are directly associated with their structural attributes, without directly contributing to binding events that result in complex formation; for example, IDRs can act as entropic springs, flexible linkers or spacers ( Tompa, 2005; van der Lee *et al.*, 2014), or fly-casting regions to capture binding partners ( Shoemaker *et al.*, 2000). Furthermore, IDRs are subject to extensive pre- and post-translational regulation to modulate protein function in response to cellular stimuli ( Bah & Forman-Kay, 2016; Csizmok & Forman-Kay, 2018; Van Roey *et al.*, 2013; Van Roey *et al.*, 2012; Weatheritt *et al.*, 2012).

As a result of the fundamental regulatory functions performed by IDRs, and the cell-state conditionality of these regulatory processes, IDRs encode many of critical steps of a protein life-cycle from the ribosome to the proteasome. Consequently, IDRs play a key role in many human diseases, including cancer (p53), Alzheimer’s disease (Aβ, Tau) or Parkinson’s disease (α-synuclein) ( Shigemitsu & Hiroaki, 2018; Uversky *et al.*, 2008) and human IDR interfaces are often mimicked by pathogens to hijack host pathways and deregulate the cell ( Davey *et al.*, 2011; Dyson & Wright, 2018; Kruse *et al.*, 2019; Via *et al.*, 2015; Xue *et al.*, 2012). Given their therapeutic relevance, IDP-mediated interactions are now seen as potential drug targets ( Corbi-Verge & Kim, 2016). Therefore, a better understanding of their structure and function will help to develop new strategies to fight human diseases.

**Figure 1.**
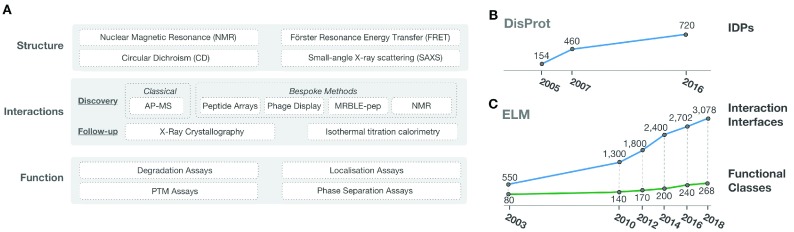
( **A**) Key experimental methods used across the three major research focuses of the IDP field: structure, interactome and function. ( **B**) The growth in structural IDP data curated by the DisProt database ( Piovesan *et al.*, 2017). DisProt was established over a decade ago in the USA, and recently brought to Europe after years of inactivity and completely re-annotated, this explains the lag in the curation. A huge amount of IDP/IDR data in the IDP literature remain uncurated and the vast majority of IDP regions remain to be characterised ( Pancsa & Tompa, 2012; Xue *et al.*, 2012). ( **C**) The growth in functional IDP data curated by the Eukaryotic Linear Motif (ELM) resource ( Gouw *et al.*, 2018). Similar to the structural IDR data, a huge amount of functional IDP/IDR data in the IDP literature remains uncurated and the vast majority of functional modules in IDPs remain to be characterised ( Tompa *et al.*, 2014).

IDP research spans several experimental fields studying protein structure and function including structural biology, biophysics, biochemistry, cell biology, proteomics, comparative genomics, systems biology, synthetic biology and pharmacology ( Figure 1A) ( Blikstad & Ivarsson, 2015; Corbi-Verge & Kim, 2016; Felli & Pierattelli, 2015; Forman-Kay & Mittag, 2013; Plitzko *et al.*, 2017). On a structural level, IDRs do not adopt a single stable highly populated structure, instead, they are structurally heterogeneous, continuously sample a wide ensemble of conformations, with preferentially sampled intramolecular contacts driving local transient secondary structure and compaction of conformations ( Davey, 2019; Dyson & Wright, 2005; Forman-Kay & Mittag, 2013; Holehouse & Pappu, 2018). Therefore, IDRs are more easily described by probabilistic models than the intuitive visual representations of structures of folded protein regions. Classical methods for structural characterisation of a protein, such as X-Ray crystallography, are unable to capture the dynamic structures of IDRs. Instead, the structural aspects of the IDRs are studied by a range of biophysical methods including Nuclear Magnetic Resonance (NMR), Small-angle X-ray scattering (SAXS), circular dichroism (CD) or Förster resonance energy transfer (FRET) ( Felli & Pierattelli, 2015; Fuertes *et al.*, 2017; Holmstrom *et al.*, 2018; Plitzko *et al.*, 2017; Tompa, 2011; Tolchard *et al.*, 2018). The results of these complementary methods can be integrated to build a model of IDR structure and dynamics.

The functional aspects of IDPs are studied by a battery of well-established cell biology and biophysical approaches ( Gibson *et al.*, 2015) ( Figure 1). Many of the approaches were developed for protein interaction elucidation, for example, by coupling mutagenesis to affinity-purification. However, interactions mediated by IDRs are often defined by their low affinity and cell-state dependent conditionality, two properties that are not always amenable to the available experimental protein interaction detection methods ( Gibson *et al.*, 2015; Van Roey *et al.*, 2014). Therefore, there are a growing number of approaches that have been specifically designed to characterise low affinity IDR-mediated interactions such as peptide arrays, proteomic phage display (ProP-PD) and peptides attached to Microspheres with Ratiometric Barcode Lanthanide Encoding (MRBLE-pep) ( Blikstad & Ivarsson, 2015; Davey *et al.*, 2017; Nguyen *et al.*, 2017; Volkmer, 2009). As IDRs often adopt a dominant conformation in their bound state, many IDR-containing interfaces can be studied in complex with the structured binding partners using structural approaches including NMR spectroscopy and X-Ray Crystallography ( Bonetti *et al.*, 2018; Fuxreiter, 2018; Iešmantavicius *et al.*, 2014; Schad *et al.*, 2018). Distinct IDR functionalities are also studied using isolation, deletion or mutagenesis of a functional module combined with bespoke assays to characterise phase separation, protein localisation, stability and post-translational modification ( Gibson *et al.*, 2015; Gouw *et al.*, 2018; Martin & Mittag, 2018).

**Figure 2.**
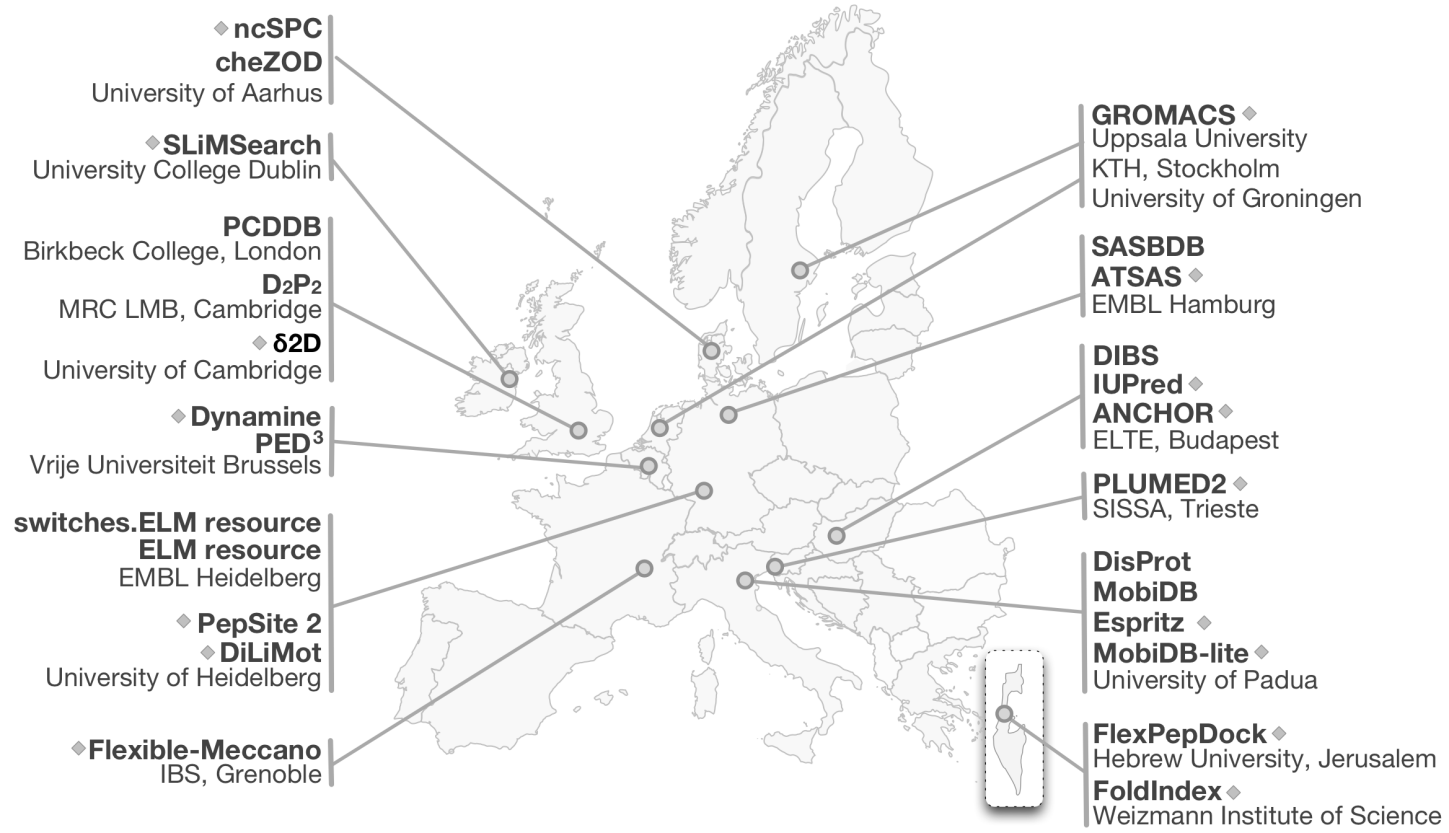
Representative core software ( Table 1, marked with a diamond) and resources ( Table 2) for the IDP field developed and hosted in Europe.

The computational IDP field tackles several distinct research tasks including: (i) the prediction of IDRs ( Cilia *et al.*, 2014; Dosztányi *et al.*, 2005), (ii) the identification of functional modules in IDRs ( Dosztányi *et al.*, 2009; Edwards & Palopoli, 2015; Krystkowiak & Davey, 2017; Neduva & Russell, 2006), (iii) docking of peptides that are intrinsically disordered in their unbound state ( Raveh *et al.*, 2011; Trabuco *et al.*, 2012), (iv) force field development and molecular simulations for IDR structure ( Best, 2017; Chong *et al.*, 2017; Huang & MacKerell, 2018; Stanley *et al.*, 2015), (v) *in silico* inhibitor design and development for IDRs ( Baggett & Nath, 2018; Santofimia-Castaño *et al.*, 2019; Yu *et al.*, 2016) and (vi) the processing of the data produced by experimental structural and functional analyses of IDRs ( Bernadó *et al.*, 2007; Franke *et al.*, 2017; Nodet *et al.*, 2009; Ozenne *et al.*, 2012) ( Table 1). The members of the European IDP community are major contributors across these computational IDP fields ( Figure 2).

**Table 1.** Representative core software of the IDP field developed and hosted in Europe.

Name	Description
IUPred	Prediction of intrinsically disordered regions. URL: *https://iupred2a.elte.hu* ( Dosztányi *et al.*, 2005)
ESpritz	Prediction of intrinsically disordered regions URL: *http://protein.bio.unipd.it/espritz/* ( Walsh *et al.*, 2012)
FoldIndex	Prediction of intrinsically disordered regions URL: *https://fold.weizmann.ac.il/fldbin/findex* ( Prilusky *et al.*, 2005)
MobiDB-lite	Prediction of intrinsically disordered regions URL: *http://protein.bio.unipd.it/mobidblite/* ( Necci *et al.*, 2017)
DynaMine	Prediction of protein backbone dynamics URL: *https://dynamine.ibsquare.be* ( Cilia *et al.*, 2014)
DiLiMot	Prediction of functional modules in intrinsically disordered regions. URL: *http://dilimot.russelllab.org/* ( Neduva & Russell, 2006)
SLiMSearch	Prediction of functional modules in intrinsically disordered regions. URL: *http://slim.ucd.ie/slimsearch/* ( Krystkowiak & Davey, 2017)
ANCHOR	Prediction of functional modules in intrinsically disordered regions. URL: *https://iupred2a.elte.hu* ( Dosztányi *et al.*, 2009)
PepSite 2	Prediction of IDP binding sites. URL: *http://pepsite2.russelllab.org/* ( Trabuco *et al.*, 2012)
FlexPepDock	Docking of IDPs to their ordered binding partner. URL: *http://flexpepdock.furmanlab.cs.huji.ac.il/* ( Raveh *et al.*, 2011)
δ2D	Secondary structure propensity from NMR data URL: *http://www-mvsoftware.ch.cam.ac.uk/* ( Camilloni *et al.*, 2012)
ncSPC	Structural propensity calculator from NMR data URL: *http://linuxnmr02.chem.rug.nl/ncSPC ( Tamiola & Mulder, 2012)*
Flexible-Meccano	Generation of ensemble descriptions of IDPs URL: *http://www.ibs.fr/research/scientific-output/software/flexible-meccano/* ( Ozenne *et al.*, 2012)
ATSAS	A data analysis suite for SAXS data including the EOM tool URL: *https://www.embl-hamburg.de/biosaxs/ ( Bernadó *et al.*, 2007; Franke *et al.*, 2017)*
GROMACS	Molecular Dynamics software applicable to IDPs URL: *http://www.gromacs.org/* ( Hess *et al.*, 2008)
PLUMED2	Library for free energy calculations URL: *https://www.plumed.org/ ( Tribello *et al.*, 2014)*

Europe hosts many of the key IDP resources ( Table 2). DisProt is the largest database of manually curated experimental data on IDP structure ( Piovesan *et al.*, 2017; Sickmeier *et al.*, 2007). MobiDB and D ^2^P ^2^ are central resources of pre-computed structural prediction of IDPs ( Piovesan *et al.*, 2018; Oates *et al.*, 2013). ELM is a manually curated database of binding regions residing in IDPs ( Gouw *et al.*, 2018). SASBDB and PCDDB are central resources for Small-angle X-ray Scattering (SAXS) and Circular Dichroism (CD) data ( Valentini *et al.*, 2015; Whitmore *et al.*, 2017). PED ^3^ is a database of conformational ensembles from Nuclear Magnetic Resonance (NMR) and SAXS data, and Molecular Dynamics (MD) simulations ( Varadi *et al.*, 2014). It is important to note that the vast majority of the computational frameworks developed by the IDP field had to be created from scratch given the unique structural and functional aspects of IDRs. For example, the analysis of IDP structure drew predominantly from polymer biophysics rather than the existing structural biology of folded proteins ( Holehouse & Pappu, 2018; Milles *et al.*, 2018; Schuler *et al.*, 2016).

**Table 2.** Representative core resources of the IDP field developed and hosted in Europe.

Name	Description
DisProt	Curated database of experimentally validated intrinsically disordered regions. URL: *http://www.disprot.org* ( Piovesan *et al.*, 2017)
MobiDB	Database of predicted intrinsically disordered regions. URL: *http://mobidb.bio.unipd.it* ( Piovesan *et al.*, 2018)
D2P2	Database of predicted intrinsically disordered regions. URL: *http://d2p2.pro/* ( Oates *et al.*, 2013)
PED3	Database of IDP conformational ensembles. URL: *http://pedb.vib.be/* ( Varadi *et al.*, 2014)
CheZOD	Database of structural propensities from NMR chemical shift data. URL: *http://www.protein-nmr.org/ ( Nielsen & Mulder, 2016)*
SASBDB	Repository for Small-angle X-ray Scattering (SAXS) data. URL: *https://www.sasbdb.org/* ( Valentini *et al.*, 2015)
PCDDB	Repository for Circular Dichroism (CD) data. URL: *http://pcddb.cryst.bbk.ac.uk/* ( Whitmore *et al.*, 2017)
ELM	Curated database of Short, Linear Motifs. URL: *http://elm.eu.org/* ( Gouw *et al.*, 2018)
DIBS	Curated database of intrinsically disordered binding regions. URL: *http://dibs.enzim.ttk.mta.hu/* ( Schad *et al.*, 2018)
Switches.ELM	Curated conditional interactomics database for IDPs. URL: *http://switches.elm.eu.org* ( Van Roey *et al.*, 2013)

The ELIXIR IDP Community has grown out of the NGP-NET COST Action (Non-globular proteins: From sequence to structure, function and application in molecular physiopathology), a scientific cooperation funded in 2015 under a Horizon 2020 EU Framework Programme. The NGP-NET community spanned 30 different countries, plus EMBL-EBI and EMBL Heidelberg. NGP-NET held a series of thematic workshops on IDPs to drive the development of computational resources and community standards. A strategic workshop titled “Intrinsically Disordered Proteins in Core Data Resources” was organised by NGP-NET at the EBI campus, Hinxton, the UK on June 1–2, 2017 to discuss the integration of IDP-related data and computational resources into the ELIXIR framework. A major outcome of this workshop was the recognition that IDP annotations are significantly underrepresented in the ELIXIR Core Data Resources (CDRs) ( Durinx *et al.*, 2016). These initial insights evolved into a comprehensive plan to develop the IDP field and integrate key IDP resources and tools into the CDRs. That plan formed the basis for the ELIXIR IDP Community proposal that was submitted and presented at the ELIXIR Head of Nodes meeting in Basel, Switzerland on September 13th, 2017. After completing the ELIXIR Community application process, including the creation of this white paper, the IDP Community proposal was accepted by the ELIXIR Head of Nodes on May 14th, 2019.

In parallel with these developments, the IDP research community has developed closer integration with ELIXIR activities. A follow up strategic meeting, “The 2 ^nd^ Workshop on Intrinsically Disordered Proteins in Core Data Resources”, was held in Prague on March 13–14, 2019 and brought together data producers, database developers and a representative of the ELIXIR Data Platform to discuss the integration of IDP data into the CDRs; and a member of the IDP research community attended the ELIXIR Interoperability Platform Face To Face Meeting on 1–2 April 2019. Furthermore, several actions have already taken place within the ELIXIR framework to tackle time-sensitive priorities including two implementation studies, “Implementation study for the integration of ELIXIR-IIB in ELIXIR Data Curation activities” and “Integration and standardisation of intrinsically disordered protein data”, focussing on interoperability. An additional, fundamental development associated with these implementation studies was the initialisation of the HUPO (Human Proteome Organisation) Proteomics Standards Initiative (PSI) Intrinsic Disorder workgroup.

The goal of the IDP Community in ELIXIR is to support the development of standards, tools and resources to accelerate the identification, analysis and functional characterisation of intrinsically disordered regions. Here, we introduce the major areas of priority for the development of an e-infrastructure that will allow the community to realise these goals while supporting the needs of the generators, users and consumers of IDP data.

## Identification of community challenges

A strategic workshop titled “An intrinsically disordered protein user community proposal for ELIXIR” was held on October 31st, 2018 at the University of Padua, Italy. The meeting was attended by participants from 13 ELIXIR nodes: Belgium, Cyprus, Czech Republic, EBI, Germany, Hungary, Ireland, Israel, Italy, Netherlands, Spain, Switzerland, and the United Kingdom. ELIXIR members in both Greece and Sweden were also interested, but could not attend. The ELIXIR Hub team was represented by John Hancock, the ELIXIR Communities and Services Coordinator and representatives from the Interoperability, Tools and Training ELIXIR platforms. Members of the 3DBioInfo and Proteomics ELIXIR Communities, the Instruct-ERIC structural biology research infrastructure, and the ELIXIR CDRs of high relevance to the IDP research, namely PDBe ( Mir *et al.*, 2018), IMEx/IntAct ( Orchard *et al.*, 2014) and UniProt ( UniProt Consortium, 2019), were also present. During the meeting, the following topics were identified as key areas of priority for the ELIXIR IDP Community.

### Area 1 – Standards and exchange formats for IDP data

The IDP field has not yet established official standards to allow consistent storage and dissemination of data. Hence, a key responsibility of the IDP Community is the definition of guidelines and standards to improve the reproducibility, interpretation, and dissemination of experimental data. The development of novel standards would result in a shift from the current “organic” interoperability of the community, where each group defines their own formats and creates a range of parsers to read the formats developed by other groups, to standardised data dissemination accessible to the larger biological community. For functional data describing protein interactions, molecular interaction interchange standards based on the guidelines of the HUPO-PSI-MI molecular standards ( Sivade Dumousseau *et al.*, 2018) can be applied. However, for structural data, no pre-existing solution exists. In the simplest cases, experimental evidence for IDRs can be mapped to protein sequences as sequence features. Structural descriptions such as IDR conformational ensembles (e.g. based on experimental NMR, SAXS or MD simulations data) or secondary structural propensities (e.g. based on NMR data) will require more descriptive standards, especially if they are probabilistic. Only a subset of the required experimental and functional ontologies are already available and most IDP data cannot be described by pre-existing terminology. IDPs will require novel entries to controlled vocabularies describing specific experimental methods and protein structural concepts.

The first step of this process was undertaken at a workshop for the ELIXIR implementation study: “Integration and standardisation of intrinsically disordered protein data” held at the University of Padua, October 29–30th, 2018. The meeting proposed a first draft Minimum Information About Disorder Experiments (MIADE) standard defining the data required for reporting an IDP experiment. A further key outcome of the meeting was the establishment of the HUPO-PSI intrinsic disorder (HUPO-PSI ID) workgroup to drive the development of the required standards, storage and dissemination formats and controlled vocabularies for the community. Upon completion, the HUPO-PSI ID recommendations will be adopted by the key community resources promoting effortless integration of IDP data into the ELIXIR CDRs. An important goal of the roadmap will be the initiation of collaborations with the experimental communities for each of the distinct structural and functional methods used in IDP research to develop: (i) experimental method specific standards and (ii) workflows with minimal required experimental detail for the characterisation of IDPs. These developments would require extensive collaboration with the data generators and the experimental method specific data repositories including SASBDB ( Valentini *et al.*, 2015), PCDDB ( Whitmore *et al.*, 2017) and the BMRB ( Ulrich *et al.*, 2008) to allow data produced by IDP researchers to be stored and disseminated in the most descriptive and efficient way possible. This will simplify the integration of the results of these analyses into the community resources. The recent development of the PDB-Dev repository of non-atomistic or part-atomistic structural data can provide a prototype for the deposition of experimental IDP data ( Vallat *et al.*, 2018; Peng *et al.*, 2019).

### Area 2 - Automated and community-driven curation

The vast majority of the experimental data describing IDPs, the functional modules encoding their function, the regulatory mechanisms conditionally controlling that function, and their dysregulation in disease, is isolated in the text of research and review articles and in poorly formatted supplementary tables. This hampers the integration of IDP information with data such as protein function, modification, splice variants and disease-causing single-nucleotide polymorphisms (SNPs). Consequently, data created at great expense is significantly underutilised. Annotation of bona fide IDPs, especially for their function, is currently a labour-intensive process. The IDP community has already been experimenting with crowdsourced curation, a topic which is of interest to the ELIXIR Data Platform, and included within Task 3 of the ELIXIR Data Platform 2019–2023 ELIXIR Programme.

DisProt is a successful example, leveraging the collective expertise of around 40 researchers from a dozen different IDP labs in as many countries. The rate and accuracy of curation can be improved by integrating the available ELIXIR e-infrastructure into the curation process through partial annotation of articles and through automatic selection, classification and prioritisation of the relevant articles for curation. For example, the annotation of IDP-mediated interactions directly into the IMEx consortium annotation portal would provide a pre-built environment for such an endeavour. Automatic triage and pre-compiling of data based on Europe PMC data would also greatly boost productivity, allowing the community to cope with the increasing amount and complexity of data being published ( Britan *et al.*, 2018). The large number of articles describing the structure and function of IDPs has highlighted the need to exploit text-mining approaches in order to eventually leverage automatic annotation from the literature. ELIXIR would facilitate integration with existing text-mining frameworks. Finally, a future goal of the community is the early capture of IDP data pre-publication directly from the data producers to reduce the need for manual curation. The coordinated experimental and computational fields within the ELIXIR IDP Community can provide a single contact point to lobby journals to require data deposition prior to publication.

### Area 3 - Integration with ELIXIR Core Data Resources

IDP annotations are significantly underrepresented in the ELIXIR CDRs and a key goal of the ELIXIR IDP community is to facilitate the integration of IDP data and services into these resources. The CDRs do not currently annotate or import experimental data describing IDR structure and function despite their high abundance and functional importance. Recently, InterPro, PDBe, and UniProt adopted IDP predictions from sequence retrieved from MobiDB, as part of an ELIXIR Implementation Study “Integration and standardisation of intrinsically disordered protein data”. This work can be used as a blueprint for the successful integration of additional IDP data into further CDRs. It would be advantageous both for the IDP Community and the CDRs to comprehensively integrate the available IDP data into these resources. Interoperability can be guaranteed by the implementation of IDP specific ontologies to describe IDP specific features and the availability of standards-compliant RESTful APIs, enabling cross-linking and programmatic access to IDP resources. Many of the key IDP resources already host RESTful APIs and utilise persistent identifiers from CDRs. However, these APIs are currently not fully interoperable with each other without bespoke adaptors, though this will be tackled by Priority Area 1 “Standards and exchange formats for IDP data”. The development of curation guidelines and standards in line with the requirements of the CDRs, or the adoption of CDR guidelines, will streamline the integration process. The IDP resources that annotate functional modules and their interactions are currently not members of the International Molecular Exchange (IMEx) consortium ( Orchard *et al.*, 2012). A key step on the roadmap is joint curation efforts between the ELIXIR IDP Community and the IMEx consortium of IDP-mediated interactions, thereby reducing duplication of effort.

### Area 4 – Standardisation, benchmarking and indexing of computational tools

The IDP research field is currently developing best practices for scientific (i/o) file formats, data analysis pipelines and benchmarking of scientific tools. These steps are being taken to raise the quality and accessibility of software developed by the community to produce more accurate, faster, more stable and user-friendly software implementation. The development and adoption of the storage and dissemination formats in *Priority Area 1* will help standardise the (i/o) file formats of IDP tools. However, several use cases will not be covered by these formats (e.g. standardised i/o formats for residue-specific IDR scoring) and additional effort will be required to formalise the output formats in these areas. Containerised software, such as those available via BioContainers, or package managers, such as BioConda, are rarely used in IDP research. However, such advances would make IDP tools more accessible to the wider community and would simplify their benchmarking.

The field of IDP research could also benefit from the development of reusable experimental workflows such as those implemented with the Common Workflow Language for commonly used IDP analysis pipelines built on top of ELIXIR CDRs and Deposition Databases. These workflows could then be managed using the Galaxy workflow manager platform. Benchmarking remains an issue for the community as the lack of common benchmarking datasets has hampered the systematic assessment of IDP tools. As a result, many publications have claimed superior performance for biased datasets, furthering the need for standardisation. Due to the availability of high-quality manually curated IDP data from DisProt, the community can provide gold standard blind datasets to run periodic benchmarking assessments of IDR prediction tools. This approach has recently been successfully applied by the Critical Assessment of Intrinsic Disorder (CAID) initiative. A similar platform for the comparison of methods predicting functional modules within IDPs or aligning homologous IDRs would also benefit the community and drive advances in these developing fields.

The development of Open Source software to benchmark IDP analysis methods across a wide range of performance metrics will simplify and standardise the assessment of IDP tools. OpenEbench can be used as an information hub to distribute reference datasets, to run comparative assessments and to publish benchmarking results. From a technical point of view, the adoption of BioContainers and/or Galaxy, and the standardisation of input and output formats would streamline the assessment process. Benchmarking results covering both scientific and technical aspects of the available IDP analysis tools can be hosted at OpenEBench to simplify method selection by users. Finally, the addition of these computational tools for the analysis of IDPs to the bio.tools registry would improve the visibility of these tools.

### Area 5 – Development of a centralised knowledge-base for IDP data

Computational IDP researchers based in Europe develop many of the tools and resources that underlie the global IDP e-infrastructure. However, these assets are currently spread over numerous institutes and universities across Europe. The development of an umbrella resource, DisProtCentral (founded June 2017), consolidating the European IDP resources and tools through a single portal will improve the accessibility of these resources for the wider biological community. The DisProtCentral consortium will provide a central hub to access high-quality curation, annotation and predictions of structural and functional information on IDPs, in addition to providing stable identifiers/URIs to describe regions of disorder within specific proteins, enabling cross-referencing and linking between resources. The consortium will include all the stakeholders in the IDP field and provide a centralised repository for the protein disorder-related databases and tools. This will future-proof these key IDP resources against issues arising from the loss of funding or change of group focus.

This initiative to produce such a centralised resource draws attention to the need for a fit-for-purpose interoperability tooling adaptor that translates across the metadata annotations of individual resources. This will allow pre-existing data from distinct resources to be integrated via normalisation functionalities such as ontology cross-referencing for data previously mapped to different ontologies/vocabularies, or ontology term mapping for those that have not been mapped to any standard terminologies. Ideally, newly generated data should be produced FAIR-at-source (Findable, Accessible, Interoperable, and Reusable) ( Wilkinson *et al.*, 2016). DisProtCentral will play a vital role in encouraging IDP data generators to follow the standardised set of best practices: identifying recommended ontologies that are universally adopted by those data generators, defining a common schema markup strategy, and validating persistent identifiers for data to be deposited into the resource. A wider goal of the DisProtCentral resource is to provide a single point of contact to promote discourse and collaboration to ensure that the needs of the IDP data generators, users and consumers are all being met by the computational IDP researchers. An important aspect of this effort will be the development of training material for the wider biological communities describing the best practices for IDP analyses.

## Alignment with ELIXIR Activities

The community challenges identified are already well-aligned with on-going ELIXIR platform goals and activities. In particular:

*Data Platform:* The task proposed in Priority Area 3 “Integration with ELIXIR Core Data Resources” aligns with the goals of the Data Platform. The community is already in the process of integrating IDP annotation into the ELIXIR CDRs as part of two ELIXIR implementation studies, “ *Implementation study for the integration of ELIXIR-IIB in ELIXIR Data Curation activities*” and “ *Integration and standardisation of intrinsically disordered protein data*”. The recent integration of MobiDB into InterPro, PDBe and UniProt can be taken as a blueprint for the further integration of IDP information into ELIXIR CDRs. Furthermore, initial discussions have taken place to plan the integration of additional sources of IDP data. A separate task, which can benefit from ongoing Data Platform activities, is the development of an IDP curation framework as described in Priority Area 2 “Automated and community-driven curation”. This aligns with ELIXIR Data Platform Task 3 “Scalable curation” of the 2019–2023 ELIXIR Programme.

The distributed community curation for IDP resources will benefit from stronger interaction with Europe PMC, with the SciLite framework providing literature mining. This is especially important where data is scattered throughout the extensive corpus of biological literature and not properly indexed. Together with an automated curation triage step selecting relevant papers, this can boost the productivity of community curation while ensuring high-quality annotation of the IDP literature. Initial efforts have started with the design of a dedicated curation-support prototype, which has been used by DisProt curators since 2018. The service capitalises on the neXtA5 platform ( Mottin *et al.*, 2017), designed by the Text Mining group of the Swiss Institute of Bioinformatics. The demonstrator ( http://candy.hesge.ch/disprotGUI/) is able to rank articles based on a scoring function, which prioritises articles with a high density of IDP-related concepts. Thanks to the curation-support tool, it will be possible to obtain a high quality curated benchmark, with the aim to evolve the current system from a triage system to a more accurate binary classifier, being able to select not only relevant papers but also to highlight short passages of text in full-text papers likely to support the expert curation. **

*Tools Platform:* The tasks proposed in Priority Area 4 “Standardisation, benchmarking and indexing of computational tools” fall under the objectives of the Tools Platform. A major goal of Priority Area 4 is to increase productivity by developing reusable experimental workflows for commonly used IDP analysis pipelines built on top of ELIXIR CDRs. The Tools Platform can advise the ELIXIR IDP Community on the development of such workflows using the Common Workflow Language in collaboration with the ELIXIR Interoperability platform. The IDP analysis tool benchmarking by the CAID initiative is complementary to the OpenEBench benchmarking platform. OpenEBench represents an information hub for the distribution of reference datasets, the application of metrics and comparative assessments and the distribution of benchmarking results. CAID can also drive prototype development for software containers and reusable experimental workflows for the community. The outcomes could then be generalised for other applications. The bio.tools service registry can be a comprehensive census of the available software for IDP analysis and the BioContainers and Bioconda platforms can assist in the generation of software containers and their registry across different technologies to facilitate access and use of software by the IDP communities and beyond.

*Interoperability Platform:* Several of the identified priorities will benefit from the input of the Interoperability Platform. The main interaction with the platform will be to develop FAIR-compliant standards and guidelines for reporting, data exchange and retrieval of data on IDP structure and function ( Wilkinson *et al.*, 2016). The recent development of a HUPO PSI-ID workgroup and a draft MIADE standard are key steps towards this goal. IDP resources themselves will benefit from becoming FAIR and adhering and contributing to the further development of standards, controlled vocabularies and ontologies. These advances are of paramount importance to improve the dissemination of IDP data. FAIR-compliant data will also aid in the FAIRification of analysis tools where reusable computational workflows can make programmatic calls to retrieve reusable data from an integrated centralised source. The Interoperability Platform service framework has already defined the various key activities to support the “FAIRification” of data which can be applied to IDP data. The effort to make IDP data FAIR can exploit the various key services that are offered by the Interoperability Platform such as those identified as recommended practices through the ELIXIR Recommended Interoperability Resources (RIRs), and the platform mission-critical initiatives (i.e., Bioschemas, and Common Workflow Language). As this is an emerging community with new data resources in development, deploying these recommended practices in new IDP databases will highlight the IDP Community as a use case example in the mission to make data *FAIR-at-source.* This work can be supported by the Interoperability Platform under their remit to provide support for interoperability for ELIXIR Communities.

*Compute Platform:* Work being carried out in the Compute Platform can be leveraged in two main ways. Community curation requires authentication for a wide range of participants, which can benefit from the federated ELIXIR AAI approach for curator login. This could also facilitate the attribution of credit to curators. A second element would be to use the ELIXIR distributed computing infrastructure including identity and access management, data integration and container deployment across a range of appropriate compute endpoints as required, both for updating large-scale databases and to run the CAID experiment. A range of new Compute Platform activities are underway to enable an integrated approach to the deployment of relevant workflows and containers. The IDP Community can take advantage of these activities to deploy IDP-related workflows and containers.

*Training Platform:* The IDP Community is focussed on the development of the next generation of IDP researchers. Extensive training has been funded and carried out by the COST Action NGP-NET and additional training schools are planned as part of the MSCA-RISE-funded IDPfun project. The Italian ELIXIR node, which has been actively providing training, will organise a yearly training course on IDP resources. The input of the ELIXIR Training Platform will be indispensable to facilitate the development of a core set of training materials, its dissemination and inclusion in ELIXIR training courses. These materials will comprise introductory and advanced lessons on databases access, *in silico* analysis of IDPs (e.g. IDP prediction, IDP docking, MD simulations and IDP-specific primary sequence analysis techniques), and computational processing of experimental data (e.g. NMR spectra, phage display). There will also be a requirement for the development of training material related to IDP interoperability to train experimentalists, software developers, database developers and curators in topics such as IDP standards, IDP controlled vocabularies and Common Workflow Language (CWL) for IDPs. This aligns with the Interoperability Platform’s training and outreach task in the 2019–2023 ELIXIR Programme to collaborate with Training Platform members to provide Interoperability training. In collaboration with the Training Platform, the IDP Community will be able to adopt and implement a wide range of best practices and guidelines available through the ELIXIR Training Toolkit, which aims to provide a comprehensive reference resource for developing training capacity, rolling out new training programs, as well as expanding existing ones. All materials and activities developed for IDP training will be shared through TeSS for further dissemination. Additionally, train-the-trainer activities will be planned to increase the size of the IDP trainers pool and build additional training capacity.

## Alignment with other communities

### Links to ELIXIR Communities

*Structural Bioinformatics (3DBioInfo):* ELIXIR *3DBioInfo* is a community of structural bioinformaticians with the remit of continuing the development of the e-infrastructure for the storage, visualisation, analysis, annotation, and prediction of the structure of biological macromolecules and complexes. The probabilistic approach to protein structure employed by the IDP researchers is highly complementary to the work of the ELIXIR *3DBioInfo* community and together, a complete structural description of proteins can be achieved. With the two communities addressing different challenges, there are numerous opportunities for synergistic connections between them, particularly in the development of structural ontologies and standards, structure boundary definition for structural studies, methods to predict IDR-globular domain interactions and whole protein structure prediction tools.

***Proteomics***. The ELIXIR Proteomics Community aspires to improve the research on proteoforms including protein forms caused by post-translational modifications (PTMs) and sequence variants. To this end, proteoform-centric annotations of proteomes are needed. This includes information on the co-occurrences of IDRs with PTMs and non-constitutive exons, and if possible, the functional outcomes of these conditional changes to the protein. Close collaboration is desirable between the communities regarding data interoperability to promote the use of IDP and proteomics data in data analysis workflows. IDP research also applies proteomics-related technologies such as ion-mobility mass spectrometry and cross-linking mass spectrometry. In these cases, close collaboration with the proteomics community within the HUPO-PSI Mass Spectrometry workgroup will support the standardisation efforts of the IDP community.

*Rare Diseases:* The overarching goal of the ELIXIR Rare Diseases Community is to create a sustainable, reusable, and interoperable infrastructure that will enable researchers to discover, access, and analyse rare disease data. A key aspect of the analysis of rare diseases data is the elucidation of the mechanism(s) by which genetic change(s) result in a diseased state. While the interpretation of the effect of disease mutations altering globular domains is well established, IDPs represent challenges in this context. Interaction and collaboration with the ELIXIR IDP Community will provide invaluable information to aid in the understanding of disease-causing variations, truncations, and/or chimeric oncogene(s) related to IDRs. The development, and dissemination of standards for IDR structural and functional data as proposed in priority area 1 are important aspects for the collaboration between the ELIXIR IDP and Rare Diseases Communities.

*Galaxy:* Galaxy is a web-based e-infrastructure for computational biomedical research. It allows users with minimal computational proficiency to run and share data analysis workflows. This promotes reproducibility and simplifies sharing of data and results. Currently, there is limited use of Galaxy by the ELIXIR IDP community. However, one of the priority areas of the roadmap, “ *Standardisation, benchmarking and indexing of computational tools”*, is the development of reusable experimental workflows for commonly used IDP analysis pipelines built on top of ELIXIR CDRs. A collaboration with the ELIXIR Galaxy Community would be highly beneficial for such an endeavour, particularly in the development of IDP pipelines using the ELIXIR scientific workflow platform.

### Links to non-ELIXIR communities

*Instruct-ERIC:* Instruct-ERIC is a European research infrastructure for structural biology that makes high-end technologies and methods available to European researchers. Similarly to the proposed ELIXIR 3DBioInfo Community, the interdependency of the research performed by Instruct-ERIC and the IDP researchers, covering the structured and unstructured parts of the proteome, allows for extensive synergies. Instruct-ERIC coordinates access to facilities which permit the structural analysis of IDPs including several centres specialised in NMR and biophysical techniques. Furthermore, Instruct-ERIC has developed a set of high-quality training courses in structural biology. Given the complementary teaching focus of the ELIXIR IDP Community and the Instruct-ERIC initiative, future training schools with contributions from both sources would be of great benefit to any participant.

*The Dark Proteome Initiative:* The Dark Proteome Initiative is a US consortium of experimentalists with the goal of fostering collaborations amongst IDP researchers and lobbying for funding to address open and biologically important questions in the IDP field. The Dark Proteome Initiative is a readymade community of world-class generators of IDP data utilising a wide range of experimental approaches allowing the ELIXIR IDP Community to access a single entity for guidance on the needs of experimental data generators. The Dark Proteome Initiative will also be able to provide invaluable advice on the detailed definitions and requirements for the development of ontologies and standards.

*PLUMED consortium:* The PLUMED consortium has been recently established as an open community including PLUMED developers, contributors and users to help to establish reproducibility, open access data, and harmonisation of the protocols for molecular dynamics simulations, free energy calculations and other simulations that can be run with the PLUMED software (PLUMED consortium, 2019).

## Conclusions

Recent years have seen a rapid growth of interest in IDPs. This has coincided with significant advances in the *in vivo, in vitro* and *in silico* methods to study the structure and function of IDPs. Unfortunately, the basic requirements for the organisation and dissemination of the tools and data produced by the field have not advanced in step with these developments. The field is on the cusp of an era where the high-throughput characterisation of the extensive intrinsically disordered regions (IDRs) of proteins is possible. This roadmap will provide the foundation that supports this data explosion and provide a solid platform for the future biological research of IDPs.

## Data availability

No data are associated with this article.
