# Supplementary material for: An intrinsically disordered proteins community for ELIXIR
Source: F1000Res. 2019 Oct 15;8:ELIXIR-1753. [Version 1] doi: 10.12688/f1000research.20136.1 (PMC6880265; doi:10.12688/f1000research.20136.1)
Supplement: Peer review history

# Peer review: Reviewer response for version 1

**DOI:** 10.5256/f1000research.22111.r55395

**Published:** 2019-11-25

**Authors:** Osman Ugur Sezerman

**Affiliations:** 1 Acıbadem University, Istanbul, Turkey

The paper describes importance of intrainsically disordered proteins with thorough set of examples with good references. It also explains computational and experimental challenges involved in IDP research.

 A good summary of available tools and platforms are also provided. Key challenges in IDP research are identified and key activities to be carried out to tackle these challenges are provided. ELIXIR community has many of the researchers working in the field within the consortium. All the needed activities align well with ELIXIR activities as well. So it is natural that ELIXIR plays a centralized role in these activities. The paper also identified key players around the world that they can collaborate with establishing a task force to tackle major challenges IDP research needs to tackle. 

 Only issue I have with the paper is the lack of focus on reproducibility issues. That is a major problem life science research is facing today. most of the experimental results are not reproducible even computational models are not reproducible resulting in waste of billions of euros spent in funding such research activities.

 They should have a special focus group within their consortium to ensure reproducibility of experimental results within IDP community (define key information needed to reproduce results, endorse use of e notebooks and use of protocols established withing the network, etc.). They should also do the same for computational models that are developed to tackle several problems related to IDP. A section added on these issues would strengthen the paper. Also if they include other initiatives focusing on reproducbility that would strengthen their task force.

I confirm that I have read this submission and believe that I have an appropriate level of expertise to confirm that it is of an acceptable scientific standard.

# Peer review: Reviewer response for version 1

**DOI:** 10.5256/f1000research.22111.r55886

**Published:** 2019-11-21

**Authors:** Julie Forman-Kay, Iva Pritisanac

**Affiliations:** 1 Molecular Medicine, Hospital for Sick Children, Toronto, ON, Canada; 2 Cell & Systems Biology, University of Toronto, Toronto, ON, Canada; 3 Biochemistry, University of Toronto, ON, Canada

There has been a significant contribution from Europe to the field of intrinsically disordered protein (IDP) research, including development of computational tools. This white paper recognizes that science and methodological development, and reports on a meeting focused on organizing the software and the software developers supporting the IDP community in Europe. While it is extremely important to provide infrastructure support for the IDP community and efforts to increase communication and usage of tools are essential, it is not clear that the significant effort to put these resources under a single umbrella and unify data formats is of value beyond that of encouraging independent development with opportunities to interact. This white paper provides a useful report on the meeting that will be of value to the IDP computational community. However, it could be improved to provide greater clarity on the proposal and some of the suggested priorities could be re-evaluated.

**Specific comments:**

1. The white paper itself is challenging to read. It is unclear who the target audience of the article is? Is it the IDP/IDR community or the ELIXIR community, or both? In the present form, the article might be confusing to both communities, as it provides a lot (at times too much) detail for some aspects, resources, and individual tools, while omitting crucial information for others. For instance, it is unclear what from the current resources would be relocated into a new ELIXIR infrastructure and what would continue to exist independently and simply being "pointed at" from a central resource? It is also unclear exactly where central data repositories and central software platforms would reside. Many presently available resources are outlined and suggested at different points, making it unclear how everything would fit together. In addition, it is unclear if there will be a central resource(s), such as DisProtCentral, and if so would this resource(s) ultimately have independent developers, next to the participating research groups.
2. Some of the software and the databases that will be the key components are listed in Figure 2. However, not all of the outlined resources are presently functional or consistently maintained. It is therefore unclear if the initiative aims at reviving some of the resources in the future, and if so, whether this be done by the individual research groups. For the present time, it would be important to include a table with links to the websites/repositories of the operational resources, and a note on the current status of all other, e.g. "not actively developed", "under development", "under restructuring", etc.
3. It would be very helpful to include schematics to help understanding of the proposed initiative and improve the flow of the article. For instance, it would be good to use flow charts to outline the hierarchy of priorities to help understand how presently available resources fit into different priority areas and what will be the first tasks undertaken by different work groups. The priorities relating to the Area 1 (“Standards and exchange formats for IDP data") would be especially important to clarify in that sense, so that the community can be informed where to look for updates on the matter and thus help aid these efforts. Finally, the history of the interactions between the IDP community and ELIXIR and the subsequent integration into the ELIXIR core could be represented with a timeline and be in a separate subsection of the article, as this presently interrupts the flow of the article.
4. It would be important to define the strategy that the community wants to undertake towards integration of resources. For instance this could be a) integrated, dedicated databases with API access that are continuously maintained for data storage; and b) open source software with modular architecture that will enable integration into, e.g., BioConda for software development. At present, the general strategy is not clear or explicit, making the article quite confusing to follow.
5. It would be highly valuable to use this article and this platform to promote open source software development and established platforms for this purpose, such as GitHub. GitHub has proven effective in countless large-scale projects and it connects thousands of developers. The comparatively very small community of IDP computational researchers would benefit by developing, integrating, and sharing their resources through GitHub. In addition, such a platform would ensure inclusiveness.
  Related to this, it is not clear how the proposed centralization will solve the major issue of long term support for software, including funding for people to continue to maintain and develop the tools. A good way to assure a project will continue is for the project source/algorithm be fully open, in a repository such as GitHub, to enable use of modular code within other projects.
6. It is stated that DisProtCentral would be used for consolidating the European IDP resources and tools. However, it has not been mentioned what is the plan (if any) for integration of non-European resources and tools? Would there be some way for developers from outside of Europe to qualify to submit or include their software and resources in DisProtCentral?
7. There is a lot of emphasis on 'interoperability', and adaptors that could be used to this end. However, apart from the simplest workflows, e.g. annotation of predicted disordered regions on a protein sequence, it is likely that the demands for the workflows will be rapidly changing in the future. Spending effort on defining formats for data is not likely to be productive since by the time these standards can be applied they are could already be outdated. Technically speaking, it is not a problem if data are used in different formats, as long as those formats are easy to parse, since writing a parser is trivial. Therefore, the emphasis should be shifted to the modular software development that would enable developers to 'mix and match' various routines and dynamically create their own workflows adapt to the task at hand. The proposal for integration into bio.tools or BioConda in this context is a very good one.
8. There are two tools that are actively maintained to current standards, GROMACS and PLUMED2, and they are not IDP specific and they run well  regardless of ELIXIR. These points should be noted. Capabilities to download the software, see the development flow and interact with the project directly are available for these tools, but it is not clear whether these principles are a general priority.

**Minor points:**

1. A full name and abbreviation for ELIXIR should be given in the abstract.
2. International Molecular Exchange (IMEx) is mentioned in the article before the abbreviation is introduced (the abbrev. is introduced later).

We confirm that we have read this submission and believe that we have an appropriate level of expertise to confirm that it is of an acceptable scientific standard.
